# Prospective observational study to evaluate treatment satisfaction and effectiveness in patients with relapsing multiple sclerosis starting cladribine tablets (CLADREAL) in Italy

**DOI:** 10.3389/fneur.2024.1379712

**Published:** 2024-04-04

**Authors:** Massimo Filippi, Laura Ferrè, Chiara Zanetta, Caterina Rizzi, Gabriella Pessina, Francesco Assogna, Maria A. Rocca

**Affiliations:** ^1^Neurology Unit, IRCCS San Raffaele Scientific Institute, Milan, Italy; ^2^Neurorehabilitation Unit, IRCCS San Raffaele Scientific Institute, Milan, Italy; ^3^Neurophysiology Service, IRCCS San Raffaele Scientific Institute, Milan, Italy; ^4^Neuroimaging Research Unit, Division of Neuroscience, IRCCS San Raffaele Scientific Institute, Milan, Italy; ^5^Vita-Salute San Raffaele University, Milan, Italy; ^6^Merck Serono S.p.A., An Affiliate of Merck KGaA, Rome, Italy

**Keywords:** relapsing–remitting multiple sclerosis, secondary progressive multiple sclerosis, disease-modifying treatment, cladribine tablets, observational study, patient-reported outcomes

## Abstract

Disease-modifying therapies (DMTs) for multiple sclerosis (MS) reduce relapse frequency, magnetic resonance imaging (MRI) activity, and slow disability progression. Numerous DMTs are approved for relapsing forms of MS although real-world data on patient-reported outcomes (PROs) and quality of life (QoL) are needed to inform treatment choice. Immune reconstitution therapy with cladribine tablets is a highly effective treatment for relapsing MS (RMS). We present the protocol for an observational study to prospectively assess the effectiveness of cladribine tablets on clinical and MRI parameters as well as on PROs, including treatment satisfaction, QoL, sleep quality, self-perceived health, fatigue, and physical function. Enrolled patients at study sites in Italy will be adults with RMS (including relapsing–remitting and active secondary progressive MS) who are either treatment naïve or have received at least one first-line disease modifying DMT or no more than one second-line DMT. The primary objective will be change in global treatment satisfaction measured with the Treatment Satisfaction Questionnaire for Medication Version 1.4 approximately 24 months after initiating cladribine tablets in patients switching from previous DMTs. Secondary objectives will include global treatment satisfaction at earlier timepoints, will comprise treatment naïve patients, and will quantify treatment effectiveness and tolerability. We will also assess relapses, disability progression, MRI activity, and other PROs at approximately 12 and 24 months. The findings will provide insight from daily clinical practice into the patient’s experience to complement data from controlled trials and inform treatment choice. EU PAS Registration Number EUPAS49334 filed 17/10/2022.

## Background

Multiple sclerosis (MS) is a chronic, autoimmune, inflammatory disease of the central nervous system (CNS) characterized by the gradual demyelination and eventual loss of myelinated axons (1, 2). The disorder affects 2.8 million people worldwide (3, 4), and over 600,000 patients in Europe (5), of which more than 110,000 are estimated to be in Italy (6). The prominent features of MS are CNS inflammation, demyelination, and neurodegeneration which can result in significant cognitive and physical disability. Multiple sclerosis can be divided into clinical phenotypes based on the course of the disease: relapsing–remitting MS (RRMS), secondary progressive MS (SPMS), and primary progressive MS (PPMS). Relapsing–remitting forms of the disease are defined by periods of new or worsening symptoms followed by periods of partial or complete recovery; it is the most common form of MS, representing approximately 80–85% of initial diagnoses (7). Secondary-progressive MS occurs when RRMS changes clinical course to involve increasing disability that is independent of clinically overt inflammation and relapse (8). Within 20 years of RRMS onset, 50% of patients are at risk of conversion to SPMS, especially if untreated (9). Together, RRMS and active SPMS – defined as patients with clinical relapse and/or signs of magnetic resonance imaging (MRI) activity – are referred to as relapsing MS (RMS) (10). Primary-progressive MS is a less common disease form characterized by progressive disability from disease onset, in the absence of relapse (11). Both progressive forms involve lower levels of diffuse inflammation and less blood–brain barrier damage than RRMS (12).

While MS is currently incurable, disease-modifying therapies (DMTs) can reduce the frequency of relapse and MRI activity, slow disability progression, and preserve quality of life (QoL). Recently, the number of DMTs available for the treatment of RRMS has greatly increased, with therapeutic options covering several mechanisms of action currently approved by the US Food and Drug Administration and the European Medicines Agency (13, 14). In Italy, a distinction is made between first-line DMTs (dimethyl fumarate, glatiramer acetate, interferon beta, teriflunomide) and second-line DMTs [sphingosine-1-phosphate receptor 1 (S1P1) inhibitors, cladribine tablets, alemtuzumab, anti-CD20 monoclonal antibodies, and natalizumab], according to their efficacy in controlling disease activity (15).

Cladribine is a synthetic deoxyadenosine analog with the ability to cross the blood–brain-barrier at 25% (16). Cladribine is taken up by lymphocytes and activated (phosphorylated) by deoxycytidine kinase, resulting in the targeting of and sustained reduction in T and B lymphocytes, which are implicated in the inflammatory processes underlying MS pathogenesis (16, 17).

Cladribine tablets are an immune reconstitution therapy that induces transient lymphocyte apoptosis and depletion, with only minimal effects on the innate immune system, followed by immune reconstitution with improved immune tolerance (18). Treatment is administered via two short courses per year of 1.75 mg/kg (cumulative dose 3.5 mg/kg) for 2 years, after which further cladribine treatment should not be required in years three and four (19). The phase III CLARITY study demonstrated that two short courses of cladribine tablets over two consecutive years significantly improved clinical and MRI outcomes, without increasing the risk of infection, including opportunistic infections other than herpes zoster, compared with placebo (20). Treatment was also associated with improved QoL over 2 years (21); lymphopenia was the most commonly reported adverse event (22, 23). The CLARITY Extension study demonstrated that the clinical benefits of two cycles of treatment are durable without further active treatment (24, 25). More than 70% of patients who received cladribine at baseline had not experienced Expanded Disability Status Scale (EDSS) progression at year five (26), and had a significantly higher prevalence of improvement at years two and five (27). In the ORACLE-MS study, cladribine tablets significantly reduced the risk of conversion to clinically definite MS in patients with a first clinical demyelinating event compared with placebo (26); in the ONWARD study, in patients with active RMS despite interferon-ß treatment, cladribine tablets reduced the annualized relapse rate when co-administered with interferon-ß (28).

Although randomized clinical trials conducted in selected MS populations under controlled conditions may not reflect routine clinical settings (29), observational MS cohort studies have confirmed the long-term effectiveness and safety of cladribine tablets in routine practice (30–35).

Qualitative data on the patient’s perception of their QoL and treatment satisfaction have gained increasing consideration in clinical research and practice (36); this is particularly true for MS patients, where long-term accumulation of physical and cognitive disability have a major impact (37, 38). The variety of DMTs now available for treating RRMS make real-world data, patient experience, and treatment satisfaction crucial for informing patient-centered treatment decisions (39, 40).

To address this data requirement, the global Patient Reported Outcomes for Multiple Sclerosis (PROMS) initiative (36, 41) promotes ‘effective patient engagement’ through the increased use of patient-reported outcome (PRO) measurements in research and routine care settings. Such intervention includes the use of a variety of validated questionnaires that allow patients to report their health status directly (42, 43); these questionnaires are useful for assessing patient perception of disease burden, and provide valuable insight into the effect of MS and its treatment on their lives (44–46). Collecting the patient’s point of view is a crucial aspect of investigating the value of a treatment. Toward this end, several ongoing studies are investigating the effect of cladribine tablets on adherence and treatment satisfaction, including the CLICK-MS (NCT03933215) and MASTER-2 (NCT03933202) studies (47), and the CLAD CROSS study (NCT04934800) (48) CLICK-MS and MASTER-2, two ongoing phase IV studies, aim to evaluate the adherence and safety of cladribine in patients switching, respectively, from injectable therapy and oral or infusional DMTs for suboptimal response. The primary outcome is ARR at 24 months (47). In the interim analysis, it was demonstrated that the adherence rates to the full first year treatment dose (1.75 mg/kg), as self-reported by patients on the MS-TAQ, were ≥ 97.2% (*n* = 35) and ≥ 96.5% (*n* = 88) among MS-TAQ respondents in CLICK-MS and MASTER-2, respectively (49). The CLAD CROSS study investigates patients previously treated with platform therapies, to analyze as primary endpoint the ARR and among secondary endpoints treatment satisfaction. The interim analysis demonstrates an increase of median TSQM v1.4 score at 12 months of 82% (48, 50).

DMTs may have properties that adversely impact treatment satisfaction, such as inconvenient administration routes and schedules, long treatment durations, and potential side effects (51). Conversely, greater satisfaction with a treatment could have a positive impact on its performance in daily life, for example, by improving adherence. Moreover, it is important to describe other dimensions of PROs, as the assessment of QoL in patients with MS. This parameter is highlighted by studies that show that any increase in QoL during treatment is accompanied by improvements in fatigue, depression, and cognition (52); the amelioration of sleep and the engagement with physical exercise can help alleviate MS symptoms. The growth in PRO assessment is expected to further increase – while clinical, MRI, and biomarker measures are essential MS outcomes, any comprehensive assessment should also include those reported by the patient (53). Patient perceptions frequently differ from those of clinicians (54), and PROs are important tools for capturing the patient’s subjective experience of the disease (55–57).

Post-hoc analysis of data from the randomized CLARITY study suggested that cladribine tablets improve QoL in patients with RMS (21). In the real-world setting, the CLEVER study conducted in patients with RMS in Germany (58), and preliminary results from the European phase IV CLARIFY-MS study in patients with highly active RMS (59), both indicate high treatment satisfaction with cladribine tablets. However, real-world data from Italy on patient satisfaction and QoL with cladribine tablets are still required as data remains scarce.

## Aims

This prospective study will assess the effectiveness of cladribine tablets on PROs and routine clinical and MRI parameters in DMT-naïve or previously treated patients with RMS in a real-world setting. Patient reported outcomes will include measures of treatment satisfaction, QoL, sleep quality, self-perceived health, fatigue, and physical function.

### Study objectives

#### Primary objective

To assess the change in global treatment satisfaction 24 months after initiating therapy with cladribine tablets in patients with RRMS switching from a first-line DMT, and patients with RMS switching from a second-line DMT.

#### Secondary objectives

- To assess the change in global treatment satisfaction at 12 and 14 months after initiating therapy with cladribine tablets in patients with RRMS switching from a first-line DMT and patients with RMS switching from a second-line DMT.
- To assess the change in global treatment satisfaction at 12, 14, and 24 months after initiating treatment with cladribine tablets in DMT-naïve patients.
- To assess the change in treatment satisfaction in terms of effectiveness, side effects, and convenience at 12, 14, and 24 months after initiating treatment with cladribine tablets in patients with RRMS switching from a first-line DMT, in patients with RMS switching from a second-line DMT, and/or DMT-naïve patients.
- To assess relapses, disability progression, MRI activity, and PROs (QoL, sleep quality, illness perception, and self-assessed physical function and fatigue) at 12 and 24 months after initiating treatment with cladribine tablets in all patient groups.

#### Exploratory objectives

- To characterize wash-out strategies for previous DMTs prior to cladribine treatment, assessed by time between DMT discontinuation and the first dose of cladribine tablets.
- To describe lymphocyte dynamics over 24 months of treatment.
- To describe ambulatory function and upper limb function over 24 months of treatment.
- To describe cognitive function over 24 months of treatment.
- To describe the safety of cladribine tablets over 24 months of treatment.

## Methods

This study was registered at the European Union electronic Register of Post-Authorisation Studies (EU PAS Register) on 17/10/2022, Registration Number EUPAS49334.^1^ This protocol conforms to Standard Protocol Items: Recommendations for International Trials (SPIRIT) recommendations.

### Observational study design

This prospective, multicenter observational study will enroll patients in three treatment groups: Group A, patients switching from first-line DMTs (platform therapies: interferon beta, teriflunomide, dimethyl fumarate, glatiramer acetate); Group B, patients switching from second-line DMTs (S1P1 inhibitor, alemtuzumab, anti-CD-20, natalizumab); and Group C, DMT-naïve patients. Cladribine tablets will be prescribed in accordance with local clinical practice and will be fully independent of the decision to enroll the patient in the study.

The baseline visit will take place within 3 months of the first dose of cladribine tablets, or within 1 month of the date of discontinuation in patients switching from a prior DMT – in patients who are treatment naïve, baseline recordings can be taken at Visit 1. The 1 month period of washout after treatment discontinuation is the one most frequently applied in clinical practice to reduce the risk of rebound.

**Figure 1:**
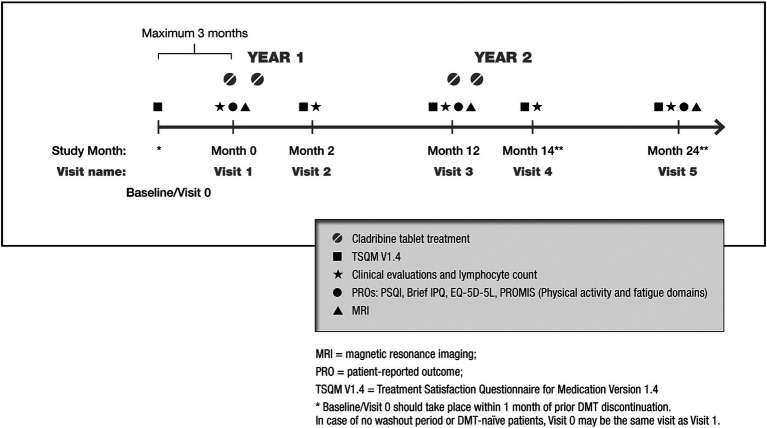
Overview of the CLADREAL study design. *Baseline/Visit 0 should take place within 1 month of prior DMT discontinuation. Visit 0 may be the same as Visit 1 when no washout is required (DMT naïve). **Visit 4 and 5 may be delayed if the second treatment cycle is delayed. Visit 4 should take place approximately 2 months after start of Year 2 treatment (up to Month 20). Visit 5 should take place approximately 12 months after start of Year 2 treatment (up to Month 30). MRI, magnetic resonance imaging; Mo., month; PRO, patient-reported outcome; TSQM V1.4, Treatment Satisfaction Questionnaire for Medication Version 1.4; IPQ, Illness Perception Questionnaire; EuroQoL EQ-5D-5L, EuroQoL 5-Dimension 5-Level; PRO, patient reported outcomes; PROMIS®, Patient-Reported Outcomes Measurement Information System; PSQI, Pittsburgh Sleep Quality Index.

Patients will be monitored according to the cladribine tablet Summary of Product Characteristics for approximately 24 months after the initial dose, or for up to 30 months if the second treatment cycle is delayed. Clinical visits will follow study-site practice and are expected at (or very near to) the first dose (‘treatment start’), and approximately 2, 12 and 14 months later (i.e., the latter being 2 months after starting the second treatment cycle); a final visit will occur 24 months after treatment start (i.e., 12 months after the start of the second treatment cycle). At the visits, assessments will be recorded as per routine clinical practice, supplemented by the above-mentioned PROs. An overview of the study design is presented in Figure 1.

Evaluation 14 months after treatment initiation will be performed because it marks the completion of the full administration course, so after the second month of the second year in accordance with Cladribine tablet dosing. This will allow assessing whether the administration schedule has an impact on treatment satisfaction.

### Study population

The study population will be recruited from approximately 45 sites in Italy, selected for regional representation. Enrolled patients will meet inclusion (Box 1) and exclusion criteria (Box 2).

**BOX 1 Inclusion criteria.**

1. Patients must read and fully understand the Informed Consent Form and must voluntarily give written informed consent.
2. Male or female patients ≥18 years old.
3. Fulfillment of the indication for treatment with cladribine tablets in accordance with the local SmPC as per standard of care. The decision to prescribe cladribine tablets by the treating physician is taken prior to and independently of the decision to enroll the patient in the study.
4. Patients who meet one of the following criteria:

- Treatment with one or more first-line DMTs (interferon beta, teriflunomide, dimethyl fumarate, glatiramer acetate) prior to initiation of cladribine tablets;
- Treatment with a maximum of one second-line DMT (S1P1 inhibitor, alemtuzumab, anti CD-20, or natalizumab) prior to initiation of cladribine tablets;
- DMTs naïve prior to initiation of cladribine tablets.

DMTs, disease modifying therapies; S1P1, sphingosine-1-phosphate receptor 1; SmPC, Summary of Product Characteristics

**BOX 2 Exclusion criteria.**

1. Patients who were treated with more than one second-line DMT prior to initiation of cladribine tablets.
2. Patients who discontinued the most recent prior DMT (if any) more than one month before enrollment.
3. Patients with a history of alcohol or drug abuse that could potentially interfere with their participation in the study.
4. Patients who have received cladribine in the past.
5. Concurrent participation in an investigational study in which patient assessment and/or treatment may be dictated by a protocol.
6. Patients who, at the discretion of the investigator, may not be able to provide reliable information for the study or are likely to be lost to follow-up during the first months of the study.

DMTs, disease modifying therapies

### Sample size

The study size was based on the global satisfaction domain of the Treatment Satisfaction Questionnaire for Medication Version 1.4 (TSQM V1.4) at Visit 5 (Month 24) in the treatment groups. Confidence intervals (CI) around the mean in each group were used to evaluate the precision of the estimates for a given sample size. In each group, a standard deviation (SD) of 18.48 (55) and a range of values for the mean global satisfaction score of 65, 70 and 75 were considered. Assuming a total enrolment of 391 patients (195 in Group A, 98 in Group B, and 98 in Group C) and a dropout rate of 15%, a total of 340 patients (170 in Group A, 85 in Group B, and 85 in Group C) was used to evaluate precision around the mean. The 2-sided 95% CIs show that sample sizes of 170 and 85 per group will provide narrow CIs around the expected mean.

### Data collection

Assessments in this non-interventional study will form part of routine clinical practice and relevant data will be collected during visits scheduled according to prescribing information (17), and as clinically indicated. Patients will attend visits at the discretion of the treating physician; therefore, the timing of the data collection is approximate. An overview of the planned data collection and order of assessments is provided in Table 1.

**Table 1:** Data collection schedule.

Assessment Approximate time since first dose (mo)	Baseline Visit 0 -3 to 0	Visit 1 0	Visit 2 2	Visit 3 12	Visit 4 14	Study end Visit 5 24
Informed consent	X					
Inclusion/exclusion criteria	X					
Demographic data	X					
Weight		X^a^		X		
Relevant medical history	X					
Comorbidities	X					
Concomitant medications	X	X	X	X	X	X
MS medication history	X					
MS disease history^b^	X					
TSQM V1.4^c^	X		X^e^	X	X^e^	X
Other PROs^d^		X		X		X
Therapy with cladribine tablets		X		X		
Relapse report		X	X	X	X	X
EDSS		X	X	X	X	X
9HPT^f^		X		X		X
T25FW^f^		X		X		X
Rao’s brief repeatable battery^f^		X		X		X
MRI data		X		X		X
Lymphocyte count		X	X^e^	X	X^e^	X
Safety recording and reporting	X	X	X	X	X	X
End of study						X

9HPT, 9-Hole Peg Test; EDSS, Disability Status Scale; Mo., month; MRI, magnetic resonance imaging; MS, multiple sclerosis; PRO, patient-reported outcome; T25FW, Timed 25-Foot Walk; TSQM V1.4, Treatment Satisfaction Questionnaire for Medication Version 1.4.

a Body weight should be assessed on cladribine tablet initiation, or nearest prior visit.

b MS disease history: number of relapses within 12 months prior to cladribine tablet initiation, and number of new/enlarged MRI lesions (T1, gadolinium enhancing (Gd+), T2 and combined unique active (CUA) lesions) measured on the last MRI performed as per normal clinical practice prior to treatment initiation.

c To be collected within 1 month of previous treatment interruption.

d Patient-Reported Outcomes Measurement Information System, Pittsburgh Sleep Quality Index, Brief Illness Perception Questionnaire, and EuroQoL 5-Dimension 5-Level questionnaire.

e If it is not possible to attend Visit 2, the TSQM V1.4 and lymphocyte count may be collected remotely.

f Upon availability, if collected as per normal clinical practice.

### Outcome measures

#### Primary outcome measure

The primary study outcome is change from baseline at Visit 5 in the global satisfaction domain of the TSQM V1.4 in patients with RRMS switching from a first-line DMT, and in patients with RMS switching from a second-line DMT.

The TSQM V1.4 (60) is a conceptually and psychometrically valid PRO instrument that measures treatment satisfaction with good psychometric measurement properties in patients with RMS (61). Its 14 items assess four key dimensions of treatment satisfaction: effectiveness (3 items), side effects (5 items), convenience (3 items), and global satisfaction (3 items). Except for ‘side effects’, which has yes/no responses, each item has either five or seven responses, scored from 1 (least satisfied) to 5/7 (most satisfied). Item scores are summed to give four domain scores, which are each in turn transformed to a scale of 0–100.

#### Secondary outcome measures

##### Treatment satisfaction

- Change from baseline in the TSQM V1.4 global satisfaction domain will be assessed separately in patients switching from first-line DMTs, and those switching from second-line DMTs at Visits 3 and 4.
- Change from baseline in the TSQM V1.4 domains of effectiveness, side effects, and convenience will be assessed at Visits 3, 4, and 5.
- Change from Visit 2 in the TSQM V1.4 global satisfaction domain at Visits 3, 4, and 5 will be assessed separately in patients switching from first-line DMTs, those switching from second-line DMTs, and DMT-naïve patients.
- Change from Visit 2 in the TSQM V1.4 domains of effectiveness, side effects, and convenience at Visits 3, 4, and 5 will be assessed separately in patients switching from first-line DMTs, those switching from second-line DMTs, and DMT-naïve patients.

Relapse will be defined as patient-reported and clinician-confirmed symptoms typical of an acute CNS inflammatory demyelinating event, developing acutely or sub acutely, with a duration of 24 h or more, in the absence of fever or infection (62). To avoid carryover or rebound effects, the analysis of time to first relapse and annualized relapse rate will exclude relapses with onset within the first 6 months after cladribine tablet initiation.

Disability progression will be assessed via the EDSS (63) at Visits 3 and 5 to determine the proportion of patients with sustained disability progression, improvement, or confirmed stability over 6 months.

The proportion of patients who remain free from MRI activity will be determined, with activity defined as T1 gadolinium enhancing (Gd+) lesions and/or new or enlarging T2 lesions. Combined unique active lesions (defined as Gd + T1, or new/enlarging T2 lesions, or both, without double counting) will be recorded at Visits 3, and 5. All scans will be performed at the discretion of the treating physician in accordance with routine clinical practice (64).

Additional PROs will be determined at baseline (Visit 0 or 1, depending upon group), Visits 3, and 5. This will include the use of:

- EuroQoL 5-Dimension 5-Level questionnaire (65).
- Sleep quality assessment via the Pittsburgh Sleep Quality Index (66); scores range from 0 to 21, with higher scores reflecting poorer night-time sleep quality (67).
- Patients’ cognitive and emotional representations of their illness as assessed by the Brief Illness Perception Questionnaire (68, 69).
- Physical function and fatigue severity assessment as determined by the Patient-Reported Outcomes Measurement Information System (PROMIS), Short Form (SF)-15, and SF-8 questionnaires (70, 71).

#### Exploratory outcome measures

- The effect of wash-out duration between prior DMT discontinuation and first dose of cladribine tablets will be determined for patients with previous exposure to DMTs, both overall and according to specific therapy.
- When laboratory services are available, lymphocyte counts will be monitored at all visits; the proportion of patients experiencing one or more lymphopenia events of any grade at any visit, and the change from baseline at Visit 5, will be assessed.
- Changes from baseline at Visits 3, and 5 in ambulatory function measured by the Timed 25-Foot Walk (72) will be assessed; the proportion of those with clinically meaningful deterioration [20% or above (73)] will be recorded.
- Changes from baseline in upper limb function will be assessed at Visits 3, and 5 using the 9-Hole Peg Test (74).
- Changes from baseline in cognitive function will be assessed at Visits 3, and 5 using Rao’s Brief Repeatable Battery, using alternative versions of the battery (A, B, A) to minimize learning effects (75, 76).
- The frequency of adverse events, serious adverse events, pre-specified potential safety events, and adverse drug reactions will be reported during the 24-month study period.

Patients may discontinue the study (i.e., withdraw consent) at any time; reasons for study discontinuation will be recorded when known. In addition, patients will be mandatorily withdrawn from the study if they do not receive the first dose of cladribine tablets within 3 months of enrolment, or they switch to another DMT after starting treatment with cladribine tablets. Patients who discontinue treatment will continue to be followed until the end of the follow-up period. A patient will be considered lost to follow-up after three documented failed attempts to contact them.

#### Data management and statistical methods

All analyses will be performed on patients who receive at least one dose of cladribine tablets following enrolment. Given the descriptive nature of the study, no formal statistical hypothesis will be tested. Descriptive statistics will be used to summarize data. Continuous variables will be summarized using the mean, standard deviation, median, first and third quartile (Q1-Q3), minimum and maximum. Categorical variables will be presented as frequency counts and percentages (n, %). Confidence intervals, if calculated, will be two-sided with a confidence probability of 95%, unless otherwise specified. For continuous data, CIs for the mean will be calculated assuming a normal distribution; those for binary outcomes will be presented using the Clopper-Pearson method. Detailed methodology for summary and statistical analyses of data collected will be documented in the integrated analysis plan. Statistical analyses will be performed using SAS (version 9.4 or higher).

## Discussion

This observational study will collect data in the context of routine clinical practice following the normal standard of care, rather than a study-mandated assessment schedule with prescribed patient visits. This approach, however, may lead to inconsistent and variable data collection across patients and study sites, with possible missing data, information bias, and residual confounding factors. In addition, potential bias may arise from patient selection (as the exclusion criteria for washout period within 1 month), patients may be lost through attrition, and variability in cladribine treatment may limit interpretation of the results (e.g., decreased adherence to, or early discontinuation of, cladribine tablet treatment courses, or potential interactions with medication for comorbidities). No statistical comparison between the different study groups is planned, because these groups are likely to be quite different. As a consequence, possible differences detected would depend from patients’ characteristics and not from treatment sequencing.

However, the strength of any observational study includes its ability to reflect normal daily clinical practice more closely than the randomized controlled trial, both in terms of the heterogeneous patient populations involved, and the medical interventions administered. In addition, the use of PROs will provide unique insight into the patient’s experience, including the impact of treatment on daily life, even if, some differences between QoL items will probably show a ceiling effect in some participants, particularly those with low levels of physical disability. Real-life observational studies are essential for improving clinical practice, and complement randomized controlled trials by providing clinically relevant, real-world data.

## Ethics statement

The studies involving humans were approved by ethics committee of IRCCS Ospedale San Raffaele. The studies were conducted in accordance with the local legislation and institutional requirements. This protocol conforms to Standard Protocol Items: Recommendations for International Trials (SPIRIT).

## Author contributions

MF: Conceptualization, Investigation, Methodology, Writing – review & editing. LF: Investigation, Methodology, Writing – review & editing. CZ: Investigation, Methodology, Writing – review & editing. CR: Conceptualization, Investigation, Methodology, Writing – original draft, Writing – review & editing. GP: Conceptualization, Formal analysis, Investigation, Methodology, Writing – original draft, Writing – review & editing. FA: Conceptualization, Writing – review & editing. MR: Conceptualization, Writing – review & editing.

## Footnotes

^1^ https://www.encepp.eu/encepp/viewResource.htm?id=49335
